# Deciphering the Influence of Electrolytes on the Energy Storage Mechanism of Vertically-Oriented Graphene Nanosheet Electrodes by Using Advanced Electrogravimetric Methods

**DOI:** 10.3390/nano10122451

**Published:** 2020-12-07

**Authors:** Tao Lé, Gérard Bidan, Florence Billon, Marc Delaunay, Jean-Michel Gérard, Hubert Perrot, Ozlem Sel, David Aradilla

**Affiliations:** 1 Laboratoire Interfaces et Systèmes Électrochimiques, LISE, UMR 8235, Sorbonne University, CNRS, 4, Place Jussieu, 75005 Paris, France; tao.louca.le@gmail.com (T.L.); florence.billon@sorbonne-universite.fr (F.B.); hubert.perrot@sorbonne-universite.fr (H.P.); 2 IRIG-SyMMES, University Grenoble Alpes, CEA, CNRS, F-38000 Grenoble, France; bidan.gerard@gmail.com; 3 IRIG-PhELIQS, University Grenoble Alpes, CEA, F-38000 Grenoble, France; marcyvesdelaunay@yahoo.fr (M.D.); jean-michel.gerard@cea.fr (J.-M.G.); 4 Institute of Inorganic Chemistry, University of Goettingen, Tammannstrasse 4, 37077 Goettingen, Germany

**Keywords:** graphene, supercapacitor, electrolyte, ionic liquids, energy storage mechanisms, EQCM, ac-electrogravimetry

## Abstract

Electrolyte composition is a crucial factor determining the capacitive properties of a supercapacitor device. However, its complex influence on the energy storage mechanisms has not yet been fully elucidated. For this purpose, in this study, the role of three different types of electrolytes based on a propylene carbonate (PC) solution containing tetrabutylammonium perchlorate (TBAClO_4_), lithium perchlorate (LiClO_4_) and butyltrimethylammonium bis(trifluoromethylsulfonyl)imide (N_1114_TFSI) ionic liquid on vertically-oriented graphene nanosheet electrodes has been investigated. Herein, in situ electrochemical quartz crystal microbalance (EQCM) and its coupling with electrochemical impedance spectroscopy (EIS), known as ac-electrogravimetry, have allowed the dynamic aspects of the (co)electroadsorption processes at the electrode-electrolyte interface to be examined. A major contribution of ClO_4_^−^ anions (TBAClO_4_) was evidenced, whereas in the PC/N_1114_TFSI mixture (50:50 wt%) both anions (TFSI^−^) and cations (N_1114_^+^) were symmetrically exchanged during cycling. In the particular case of LiClO_4_, solvation of Li^+^ cations in PC was involved, affecting the kinetics of electroadsorption. These results demonstrate the suitability of dynamic electrogravimetric methods to unveil the interfacial exchange properties of mobile species for the conception of new high performance energy storage devices.

## 1. Introduction

In recent years, the emerging miniaturization technologies have transformed key manufacturing and processing concepts to design an unlimited range of new products by leveraging skills from across many domains, conceiving new product–market paradigms and future innovative products ranging from biomedicine (biomedical implants), robotics, and smart watches to wireless sensors. One of the most widespread representative examples is at present known by the term “The Internet of Things”, which is a revolutionary and trendy concept to describe the exchange of data between portable, smart and connected devices [1]. In this scenario, equipment is becoming more digitized and connected, establishing networks between machines, humans, and the internet, leading to the creation of new ecosystems that enable higher productivity, better energy efficiency, and higher profitability [2,3]. Consequently, the need to find reliable self-powered and self-sustaining autonomous micro-power units appears critical to accomplish tomorrow’s technical challenges in the field of portable energy storage. Within this context, microbatteries represent the most common choice but they still exhibit major concerns due to their limited lifetime and low power density. In recent years, micro-supercapacitors (MSCs) have attracted a great deal of attention owing to their outstanding properties in terms of high power density (>10 mW cm^−2^), extraordinary cycling stability (>100,000 cycles), excellent reversibility (~99% coulombic efficiency) as well as an ultra-fast discharge rate (ms), which make them a promising candidate compared to batteries [4,5]. However, finding a mature solution concerning the research of nano-hierarchized robust materials is of vital importance in order to comply with the strict performance requirements for MSC commercialization [6]. Tremendous efforts have been devoted to date, mainly to investigate a large variety of carbonaceous nanostructures such as carbon nanotubes, diamond, onion-like carbon, activated carbon [7] and more recently, transition metal carbides, as for example MXenes [8], silicon nanostructures (e.g., silicon nanowires and derivatives) [9], metal dichalcogenides [10] or pseudocapacitive materials including transition metal oxides, electroactive conducting polymers or nitrides [11]. Among them, graphene-based materials have attracted a great deal of attention in this domain owing to their great specific surface area (up to 2630 m^2^ g^−1^), leading to interesting areal capacitances (>2 mF cm^−2^) [12,13,14]. More specifically, in the particular case of graphene derivatives, vertically oriented graphene nanosheets (VOGNs) have awakened a special interest in the field of supercapacitors due to their peculiar properties compared to horizontal graphene. Thus, VOGNs exhibit a non-stacking morphology characterized by the presence of self-organized and interconnected channels perpendicular to the substrate, which favor ion diffusion and wettability of the electrolyte. In addition, their high rigidity and 3D inter-network structure leads to a large accessible surface area and high in-plane conductivity [15]. In this scenario, numerous reviews [15,16,17,18] and chapters of books [19,20] dealing with VOGNs and their application for electrochemical energy storage devices have already been reported, demonstrating their enormous potential for MSC devices. In spite of the important progress conducted in this technological area, their electrochemical behavior has not yet been fully understood.

Over the past years, unraveling the phenomena and chemical-physical processes involved at the electrode–electrolyte interface during the charge–discharge cycles of a supercapacitor is key to understand its electrochemical performance [21,22]. In this line, advanced modelling techniques based on molecular dynamics and numerical simulation [23,24,25,26] and in situ experimental techniques [27,28] have already provided important insights on the comprehension of energy storage mechanisms in supercapacitors. From the experimental point of view, numerous analytical techniques based mainly on nuclear magnetic resonance (NMR) spectroscopy [29,30,31], coupled with electrochemical dilatometry [32] and electrochemical quartz crystal microbalance (EQCM) [33] as well as electron paramagnetic resonance [34], EQCM [35,36,37,38,39] or small angle scattering techniques (e.g., small angle neutron scattering or small angle X-ray scattering) [40] have been employed in the field of carbon-based supercapacitors. Among them, EQCM is a powerful tool able to quantitatively measure small mass changes occurring at the electrode surface during an electrochemical process and correlate them to the electrical response. Thus, EQCM provided useful information, as for example, dealing with the effects and role of (de)solvation, electro-adsorption/desorption of both anions and cations and its ionic exchange behavior on nano/micro-porous carbon during the charge–discharge cycles. To date, EQCM has been widely employed for thin planar films based on carbonaceous structures such as carbon nanotubes or nanoporous carbon [41], pseudocapacitive materials such as transition metal oxides (MnO_2_) [42,43] or nitrides (e.g. vanadium nitrides) [44]. However, the potential of this technique has been scarcely investigated for vertical capacitive nanostructures. Within this context, we have recently reported pioneer studies dealing with the comprehension of energy storage mechanisms of capacitive vertically-aligned nanostructures such as VOGNs [45] and silicon nanowires (SiNWs) [46], or pseudocapacitive materials, as for example poly(3,4-ethylenedioxythiophene) (PEDOT) nanowires, [47] by using the EQCM and associated techniques. In the particular case of VOGNs, in the presence of a propylene carbonate (PC) solution containing tetrabutylammonium tetrafluoroborate (TBABF_4_), classical EQCM and its complementary counterpart ac-electrogravimetry (coupling of QCM and EIS technique developed by Gabrielli et al. [48]) confirmed that BF_4_^−^ anions are the major energy storage vector with high kinetics of interfacial transfer values and low transfer resistance, while cations and free solvent molecules provide non-negligible supporting roles [45]. In this study, we take a step forward to provide a complete analysis of the role of different organic and organic/ionic liquid mixture electrolytes (e.g., free solvent, anions, cations and solvated ions), the kinetics of species transferred at the electrode/electrolyte interface and their impact on the capacitive properties of VOGNs through the EQCM and associated methods.

## 2. Materials and Methods

### 2.1. Materials and Reagents

Anhydrous propylene carbonate (PC), tetrabutylammonium tetrafluoroborate (TBABF_4_), tetrabutylammonium perchlorate (TBAClO_4_), lithium perchlorate (LiClO_4_) and butyltrimethylammonium bis(trifluoromethylsulfonyl)imide (N_1114_TFSI) were respectively purchased from Sigma Aldrich and IOLITEC (Ionic Liquids Technologies GmbH, Heilbronn, Germany) and used without further purification. The water content of the ionic liquid was determined by Karl-Fischer titration (42 ppm). GaPO_4_ plain crystal resonators with a diameter of 14 mm were purchased from AWS Sensors, Valencia, Spain. After sputtering a titanium adhesion layer (~10 nm thick), gold patterns (~100 nm thick) were deposited on the resonator by evaporation. Non-aqueous reference electrode (Ag/Ag^+^ silver wire in a solution containing PC/0.1 M TBAClO_4_/0.01 M AgNO_3_, Ref. RE-7S) was purchased from ALS Co., Ltd., Tokyo, Japan.

### 2.2. Growth of VOGNs

VOGNs were grown by the electron cyclotron resonance-plasma enhanced chemical vapor deposition (ECR-CVD) technique on GaPO_4_ piezoelectric crystal resonators covered with two gold electrodes. The ECR-CVD reactor consists of a 2.45 GHz microwave power injection, two permanent magnets providing the required field for electron resonance and plasma confinement, a gas inlet and a substrate heater, which was built at CEA-Grenoble and patented by Delaunay and co-workers [49]. The experimental conditions of VOGN growth were already described in our previously reported works [45,50]. Briefly, 50 sccm C_2_H_4_ gas flow is injected into the chamber at a temperature of 480 °C using a microwave power of 280 W at a pressure of 3·10^−4^ mbar. The growth time was set to 80 min.

### 2.3. Morphological Characterization

The surface morphology of VOGN film on gold patterned GaPO_4_ crystals was examined by scanning electron microscopy (SEM, Zeiss Ultra-55, Jena, Germany) at an accelerating voltage of 10 kV at a tilt angle of 45°. The sample was fixed on a stainless-steel sample holder by using sticky carbon tape.

### 2.4. Electrochemical Characterization

A 3-electrode electrochemical cell was assembled in a glovebox under argon atmosphere at room temperature using VOGN-coated GaPO_4_ substrate as the working electrode (0.4 cm^2^ is exposed to the electrolyte), Pt wire as the counter electrode and an Ag/Ag^+^ non-aqueous reference electrode. Two PC solutions containing 0.5 M TBAClO_4_ and 0.5 M LiClO_4_, respectively, and a 50:50 wt% solution of PC and N_1114_TFSI were employed as the corresponding electrolytes. The optimal PC/N_1114_TFSI mixed solution was chosen based on its excellent electrochemical performance for supercapacitor applications as reported previously [51]. Cyclic voltammetry (CV) measurements were conducted in a potential window from −1.5 to 1 V (Δ*V*: 2.5 V) under a scan rate of 100 mV s^−1^ for each experiment. CVs were acquired by scanning the cell from open-circuit voltage (OCV ~0.1 V) from negative to positive terminal potentials. Before electrochemical tests were carried out, each test cell was cycled at a scan rate of 100 mV s^−1^ 20 times to ensure the wettability of the electrode and the equilibrium state of electrolyte ions on the graphene electrode surface. No significant change was observed during the cycling of VOGN electrodes regarding their capacitive properties. The areal capacitance (*AC*) was calculated from the CV curve by using the following equation: *AC = Q*/(Δ*V × A*), where *Q* is the average voltammetric charge, which is determined by integrating either the anodic and cathodic scans of the corresponding CV curve, and *A* corresponds to the geometric surface area of the electrode (0.4 cm^2^).

### 2.5. Electrogravimetric Analysis

A lab-made QCM device based on a Miller oscillatory circuit was used to measure the resonant frequency variation Δ*f* of the GaPO_4_ crystal (around 6 MHz), which was then converted into mass changes Δ*m* using the Sauerbrey equation [52]: $\Delta f=-k_{s}\times \Delta m$ where *k_s_* is the sensitivity factor (theoretical value: 7.92 × 10^7^ Hz g^−1^ cm^2^) [53]. According to the Sauerbrey’s equation a mass loading of 23 µg cm^−2^ was calculated, measuring the resonant peak frequency (*f*) before and after VOGN deposition in air. The *f* response of the VOGN coated resonator was monitored during electrochemical tests and more quantitative information from the EQCM data was obtained with further analysis by means of the mass per mole of electron (MPE) estimation. This analysis provides the mass/charge ratio values as a function of applied potential during a CV scan, indicating the apparent mass of the ions involved in the charge compensation. MPE can thus be expressed according to the following equation: MPE = *F* × (Δ*m*/Δ*q*), where *F* is Faraday’s constant, and ∆*m* and ∆*q* are obtained from the QCM and CV data, respectively, determined from the oxidation or reduction scan directions [54]. To check the gravimetric regime an Agilent 4294A impedance analyzer was used to perform electroacoustic impedance measurements to justify the use of the Sauerbrey equation [55]. It permitted the resonant peak frequency (*f*), the motional resistance (*Rm*) and the resonant peak width (*W*) to be measured for each resonator and following the different configurations (with(out) loadings in air or in electrolyte). Moreover, the quality factor (*Q*), *Q = f/W*, where *f* is the resonant frequency and *W* is the full-width at half-height of the resonance peak [36,37] is estimated for the resonators used in this work.

Ac-electrogravimetry was performed for 11 potentials ranging from −1.5 to 1 V (V vs. Ag/Ag^+^) using a four-channel frequency response analyzer (FRA, Solartron 1254, Solartron Analytical, Elancourt, France) and a lab-made potentiostat (SOTELEM-PGSTAT). The working electrode was polarized at a selected potential and a sinusoidal small amplitude potential perturbation (80 mV rms) was superimposed. The microbalance frequency change corresponding to the mass response was measured simultaneously with the ac response of the electrochemical systems. The resulting signals were sent to a four−channel FRA, which allowed the electrogravimetric transfer function (TF) Δm/ΔE(ω), and the electrochemical impedance ΔE/ΔI(ω), to be simultaneously obtained at a given potential under frequency modulation between 63 kHz and 10 mHz [56,57].

## 3. Results

### 3.1. VOGNs

**Figure 1:**
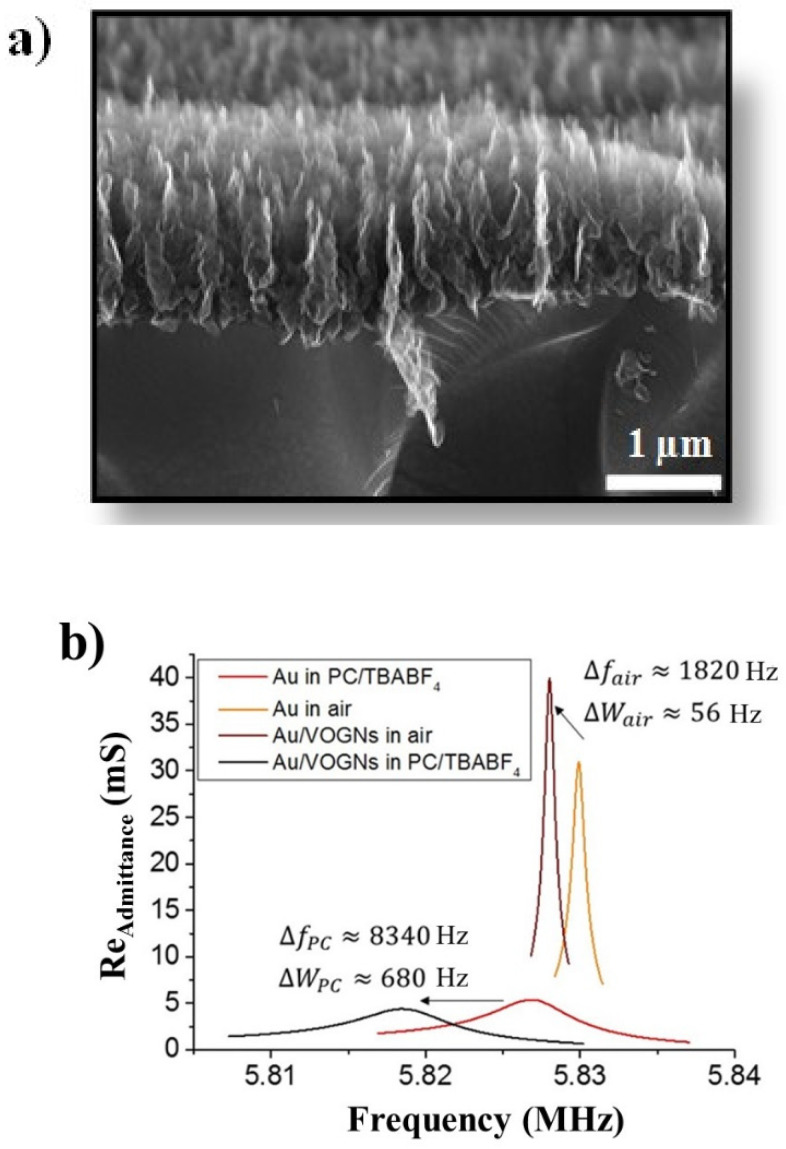
(**a**) Scanning electron microscopy (SEM) cross-sectional view of vertically oriented graphene nanosheets (VOGNs) and (**b**) the real part of the electrical admittance (*G*, mS) of the GaPO_4_ electrode at resonance frequency before growth in air (orange) and in propylene carbonate (PC) (red), and after VOGN growth in air (brown) and in PC (black).

The surface morphology of ECR-CVD grown VOGNs on GaPO_4_ substrates was examined by SEM as illustrated in Figure 1a. An interconnected non-agglomerated porous network of graphene in a vertical orientation is observed. A detailed structural, morphological and physical characterization of VOGNs based on X-ray photoelectron spectroscopy (XPS), Raman spectroscopy, transmission electron microscopy (TEM) or 4-probe conductivity techniques were already published in our previous work [51]. Consequently, a brief morphological characterization based exclusively on SEM has been reported in this work. According to the experimental conditions of the VOGN growth, previously described in the Materials and Methods Section, a total mass of 23 µg cm^−2^ with a thickness of approximately 1 µm was obtained (Figure 1a). The employment of vertical nanostructured materials for electrogravimetric measurements is highly sensitive due to hydrodynamic damping effects and viscoelastic property changes in the presence of an electrolyte, which has to be analyzed before subjecting the nanostructured materials to an electrogravimetric study. In order to understand such phenomena, electroacoustic admittance measurements have been conducted. Figure 1b displays the real part of the electrical admittance (*G*) of the resonator near the resonant frequency in four different configurations: before VOGN growth with and without PC based electrolyte, and after VOGN growth with and without electrolyte. For each condition, the resonant peak frequency (*f*), the motional resistance (*Rm*) and the resonant peak width (*W*) are directly measured. The quality factor (*Q*) was then calculated, as depicted in Table 1. These factors permit the characteristics of the resonators to be evaluated to verify the applicability of the gravimetric regime.

First, small *Rm* values are measured in air before and after VOGN deposition; the changes in *Rm* after VOGN deposition are within the standard deviation. This result confirms the rigid behavior of the VOGN materials as already demonstrated [45]. The small peak width changes (Δ*W* ~56 Hz) with respect to the loading, Δ*f* ~1820 Hz is also observed. These parameters can also be presented as the quality factor (*Q*) which are quite high (before and after VOGN deposition; measured in air). Overall, this analysis indicates the rigidity of the VOGN layer and it behaves as an extension of the GaPO_4_ resonator in air. The same comparison between blank resonators to VOGN covered resonators at OCV in the electrolyte was done: both Δ*f* and Δ*W* show a change of about 10% which leads to small changes of the quality factor. This result is attributed to the increased rugosity of the electrode surface (Au versus VOGN-modified Au electrode of the resonator). However, it is assumed that the rugosity of the rigid VOGN electrode and the viscosity of the electrolyte do not significantly change during charge/discharge of the electrode. Therefore, the influence of the surface roughness on the EQCM measurements is considered negligible [58] and VOGN-based GaPO_4_ resonators are deemed suitable for electrogravimetric measurements in PC-based electrolytes.

### 3.2. Electrochemical Performance and EQCM Results

**Figure 2:**
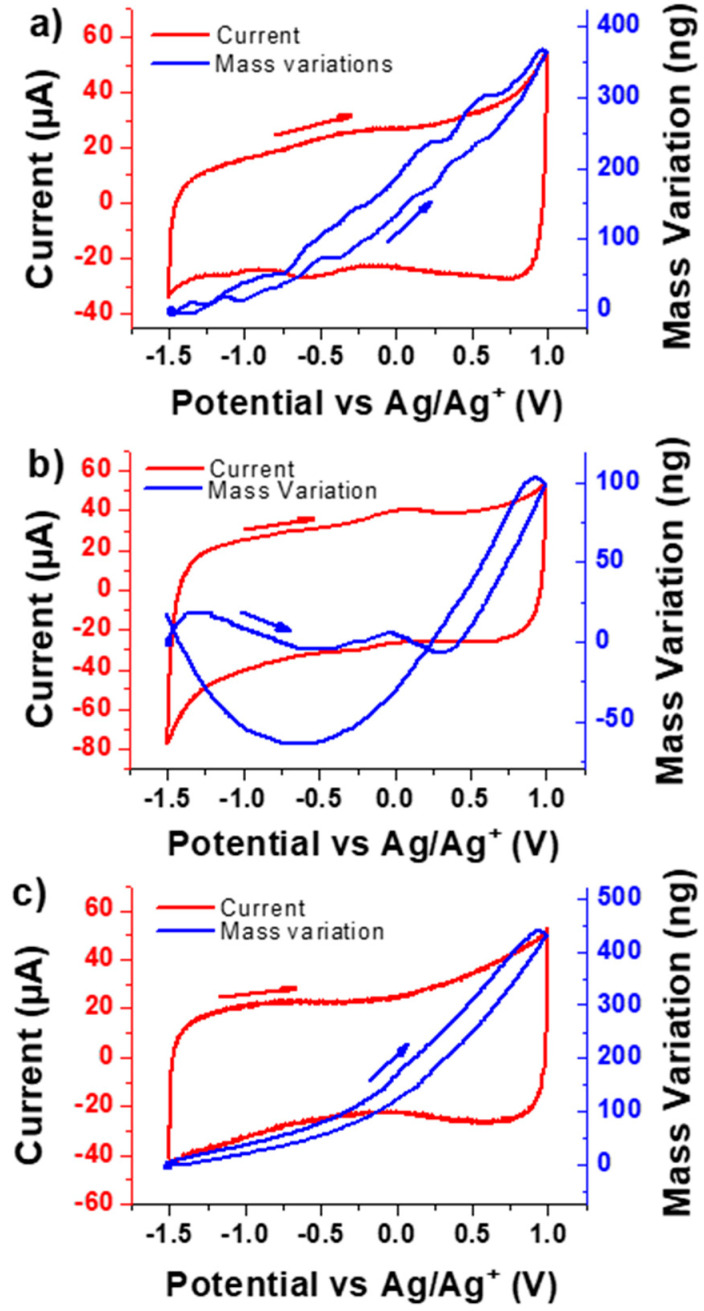
Cyclic voltammetry (CV) curves (red line) coupled with the mass measurement (blue line) on VOGN electrodes in the presence of various electrolytes: (**a**) PC/TBAClO_4_, (**b**) PC/LiClO_4_ and (**c**) PC/N_1114_TFSI at a scan rate of 100 mV s^−1^ (5th cycle is shown). Arrows indicate the scan direction. The mass variations start from the most cathodic potential, a blue point indicating clearly this relative beginning of the mass measurements.

Figure 2 displays the CV curves of the VOGN film electrodes in presence of a PC solution containing (a) TBAClO_4_, (b) LiClO_4_ and (c) N_1114_TFSI, at a scan rate of 100 mV s^−1^. As can be seen, all the CV curves show a similar electrochemical response characterized by an electrochemical double layer capacitive behavior, reflecting that the charge storage is predominantly due to the reversible electroadsorption/electrodesorption of the electrolyte ions during the charge–discharge scan. It is worth noting that the slight distortion of the voltammogram is mainly due to the presence of functional groups on the graphene surface, as reported in previous works [45,59]. As a result, quasi-rectangular shaped CV curves led to similar AC values of 1.3, 1.2 and 1.1 mF cm^−2^ for TBAClO_4_, LiClO_4_ and N_1114_TFSI electrolytes, respectively. These values are significantly higher than the ones measured for SCs based on VOGN electrodes in the presence of aqueous electrolytes of which values at 100 mV s^−1^ are 43.8, 197 and 188.8 µF cm^−2^ for Na_2_SO_4_, KOH and H_2_SO_4_, respectively [59].

In the EQCM measurements, from a qualitative point of view, a positive and a negative slope of the mass response corresponds to the contributions of an anion and a cation transfer, respectively. In Figure 2a,c, the main contribution is due to an anion and in Figure 2b, two different slopes are observed, which corresponds to a mixed contribution. In the cases where only one species is exchanged, then the MPE corresponds to its molar mass. The positive and negative values of the MPE correspond to a major contribution to the energy storage mechanism by anions and cations, respectively. VOGNs/TBAClO_4_ (Figure 2a) show a reversible mass increase of 350 ng from −1.5V to 1V vs. Ag/Ag^+^, suggesting that ClO_4_^−^ anions are predominantly exchanged with the electrode. A slight decrease in the mass variation slope below −1V vs. Ag/Ag^+^ suggests that cations are likely to play a role in this potential region. The MPE values are approximately around 70 g mol^−1^ above −1V and around 30 g mol^−1^ below −1V vs. Ag/Ag^+^ compared with the ClO_4_^−^ anion mass of 99.4 g mol^−1^ (Table 2). Positive MPE values are characteristic for the anion contribution in EQCM analyses. Therefore, anions seem to be predominant in the entire potential window, while coexisting cation exchange (permselectivity failure) or solvent contribution is suspected, especially at lower potentials due to the positive but lower MPE values in this region. A similar tendency was found in our previous work dealing with PC/TBABF_4_ [45]. A smaller cation, Li^+^, was employed in this study to investigate its effect on the energy storage mechanisms of VOGN electrodes. Figure 2b depicts the experimental results for PC/LiClO_4_. A reversible mass increase of around 150 ng was observed from −0.6 to 1 V vs. Ag/Ag^+^, showing the contribution of anions in this region with a possible contribution of the solvent. However, a reversible mass decrease of 80 ng is also observed from −1.5 to −0.6 V, reflecting that Li^+^ cations play a more important role compared to the TBA^+^ cation for the energy storage mechanisms of VOGNs. The MPE calculations resulted in around 34 g mol^−1^ above −0.5 V and −31 g mol^−1^ below −0.75 V (Table 2). These values are respectively smaller than the ClO_4_^−^ molar mass (99.4 g mol^−1^) and higher than the Li^+^ molar mass (6.9 g mol^−1^), indicating that anions and cations are not being exclusively exchanged in the entire potential window. One of the main reasons of this phenomena is attributed to the solvation of Li^+^ cations within the PC-based electrolyte. Li^+^-(PC)*_n_* solvation structures have been reported in bulk PC with 1 ≤ *n* ≤ 4 varying with salt concentration [60]. Upon Li^+^ adsorption at the VOGN surface, partial desolvation of the cations occurs and PC molecules can leave the surface due to steric hindrance. The global contribution can appear as an anion response which can indicate a more complex interpretation of the EQCM measurements [43]. The potential-dependent and time-dependent behaviors of MPE values in CV demonstrate a multi-species transfer and complex interfacial processes, which cannot be interpreted unambiguously by conventional EQCM analysis. In this line, ac-electrogravimetry results will be analyzed in the next section to provide a clearer comprehension of the charge compensation behavior at the VOGN electrode/electrolyte interface. In addition to studying the effect of cation size, the complexity degree of the electrolyte composition has also been increased and Figure 2c presents the EQCM results with PC/N_1114_TFSI as the electrolyte. A reversible mass increase of 430 ng is obtained over the whole potential window from −1.5 V to 1 V vs. Ag/Ag^+^, suggesting again that anions contribute to the majority of the ionic exchanges. The slope of this mass increase varies from negative to positive potentials, leading to MPE values around 25 g mol^−1^ below −0.3 V and 90 g mol^−1^ above 0 V vs. Ag/Ag^+^ (Table 2). TFSI^−^ anions have a molar mass of 280.1 g mol^−1^, which is much higher than the MPE values. This tendency reflects that anions are not the only species exchanged with the electrode during cycling. Under these circumstances, classical EQCM reaches its limitations and the deconvolution of the global electrogravimetric response into gravimetric and temporal components is necessary to discriminate the different species that are involved. This can be achieved by conducting ac-electrogravimetry, as a complementary tool to EQCM.

### 3.3. Ac-Electrogravimetry

**Figure 3:**
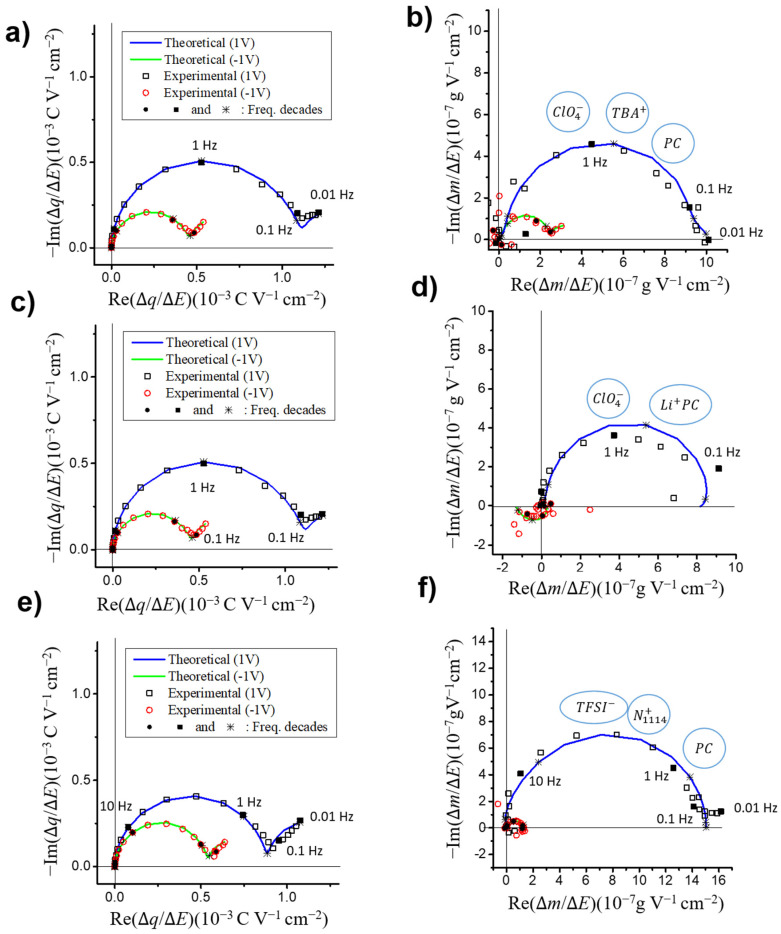
Experimental results of ac-electrogravimetry on VOGN electrodes at 1 and −1 V (squares) and their corresponding theoretical fits (lines) in Nyquist plots of the charge/potential TFs ∆*q*/∆*E*(*ω*) (**a**,**c**,**e**), and the mass/potential TFs ∆*m*/∆*E*(*ω*) (**b**,**d**,**f**) in (**a**,**b**) PC/TBAClO_4_, (**c**,**d**) PC/LiClO_4_ and (**e**,**f**) PC/N_1114_TFSI.

Ac-electrogravimetric measurements were performed on the same VOGN electrodes with the same electrolytes to better understand the contribution and dynamics of each species in the charge compensation process observed in EQCM (Figure 2). These measurements were performed at every 0.2 V from −1.5 V to 1 V vs. Ag/Ag^+^. Following each measurement, the theoretical ac-electrogravimetric models were adapted to the particular experimental cases in order to fit the impedance, charge/potential, mass/potential as well as partial mass/potential TFs with the same set of parameters. For each set of experimental data, three species were initially considered to be exchanged at the electrode/electrolyte interface: cations, anions and free solvent molecules. The species which do not participate in these exchanges were then removed from the model. The results are depicted at 1 V and −1 V vs. Ag/Ag^+^ in the form of Nyquist plots of the experimental charge/potential TF $\frac{\Delta q}{\Delta E}\left(\omega \right)$ (Figure 3a,c,e) and mass/potential TF $\frac{\Delta m}{\Delta E}\left(\omega \right)$, (Figure 3b,d,f) together with the theoretical curves fitting the experimental data. The following equation was used to fit the charge/potential TF [43]:

(1) $$\frac{\Delta q}{\Delta E}(\omega )=Fd_{f}\sum_{i}\frac{G_{i}}{j\omega d_{f}+K_{i}}\;(i:\;\mathrm{ions})$$

where *K_i_* represents the kinetics and *G_i_* describes the ease/difficulty of each species’ transfer at the electrode/electrolyte interface (*d*_f_ is the film thickness, *ω* = 2π*f* and *F* is Faraday’s constant). The mass/potential TF, Δ*m*/Δ*E*(*ω*) envelops the transfer of different species in terms of quantity (as well as in terms of the frequency domain where each process occurs). Two parameters (*K_i_* and *G_i_*) previously obtained from the charge/potential TF for each ionic species were used for fitting the Δ*m*/Δ*E*(*ω*) response with the equation below [43]:

(2) $$\frac{\Delta m}{\Delta E}(\omega )=-d_{f}\sum_{i}M_{i}\frac{G_{i}}{j\omega d_{f}+K_{i}}\;(i:\;\mathrm{ions}\;\mathrm{and}\;\mathrm{neutral}\;\mathrm{species})$$

For all electrolytes except PC/LiClO_4_ and at all potentials, a suppressed loop is observed in the upper-right quadrant of the mass/potential TF $\frac{\Delta m}{\Delta E}\left(\omega \right)$, indicating a major anion contribution. This result is in good agreement with the EQCM measurements performed in the previous section. However, for each of those cases a model using only anion exchanges would result in a larger loop for $\frac{\Delta m}{\Delta E}\left(\omega \right)$ and would not fit the experimental data. In the particular case of PC/LiClO_4_ the same reasoning applies over −0.6 V vs. Ag/Ag^+^, but below this potential the loop shifts to the lower-left quadrant, indicating a major cation contribution. The width of these suppressed loops are proportional to the mass change involved in the exchange, explaining the fact that Li^+^ exchanges observed at −1 V vs. Ag/Ag^+^ induce a much smaller loop than ClO_4_^−^ exchanges at 1 V (Figure 3c,d). However, in the case of PC/TBAClO_4_, the results were fitted with TBA^+^ cations, ClO_4_^−^ anions and free PC solvent molecules for all potentials except at 0 V vs. Ag/Ag^+^, where cations were not included in the model (Figure 3a,b). During the search for a proper theoretical model to interpret the results obtained in PC/LiClO_4_, multiple solvation numbers for Li^+^ cations have been tested. Monosolvated Li^+^ cations (PC-Li^+^) and ClO_4_^−^ anions were the only two species that resulted in good agreement of the experimental and theoretical curves (Figure 3c,d). Therefore, Li^+^ cations are found to keep a solvation number of *n* = 1 upon adsorption at the VOGN surface, probably in agreement with its small ion size and its difficulty to desolvate. Below 0 V vs. Ag/Ag^+^ the results obtained with PC/N_1114_TFSI were fitted with the participation of N_1114_^+^ cations and TFSI^−^ anions only, with an addition of PC molecules to the model above 0 V (Figure 3e,f shows only the results obtained at 1 V and −1 V as examples).

#### 3.3.1. Partial Mass/Potential TFs-Separating Each Species Contribution

**Figure 4:**
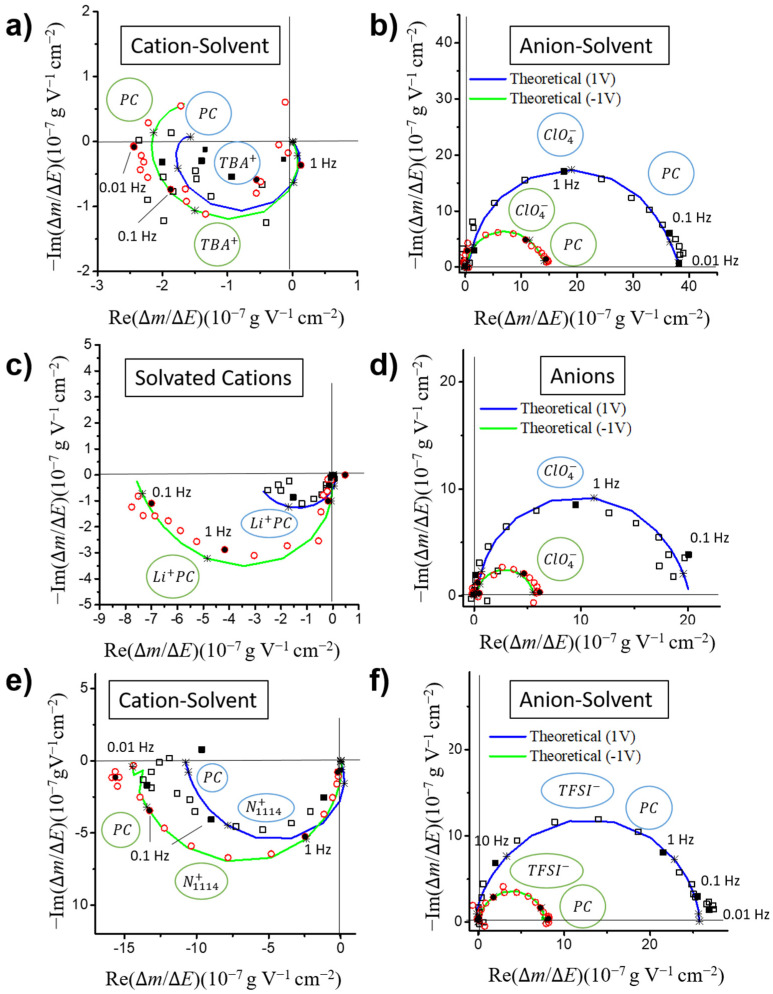
Nyquist plots of the cation–solvent (a, c, e) and anion–solvent partial mass/potential TFs (b, d, f) in (**a**,**b**) PC/TBAClO_4_, (**c**,**d**) PC/LiClO_4_ and (**e**,**f**) PC/N_1114_TFSI.

In the majority of these experiments, anions and cations are exchanged almost simultaneously and their contributions are observed at around the same frequencies, leading to only one loop with a reduced width due to the combination of both exchange mechanisms. The theoretical expressions in the ac-electrogravimetry model can be used to separate these contributions mathematically and clearly show the contribution of each species in partial TF graphs. Thus, the presence of two different species estimated by simulating the experimental data was further confirmed by carefully analyzing the partial electrogravimetric TF by removing the anion or by removing the cation contributions. Figure 4a,c,e depict the Nyquist plots of the cation–solvent partial mass/potential TFs at 1 V and −1 V vs. Ag/Ag^+^ for all electrolytes, calculated with the removal of the anion contribution from $\frac{\Delta m}{\Delta E}\left(\omega \right)$ [54,55,61]. Almost all the resulting plots only show a suppressed loop in the lower-left quadrant, characteristic for cation contribution. This crosscheck process allows our assumption of a multi ion contribution to be validated. Similarly, Figure 4b,d,f display the Nyquist plots for the anion–solvent partial mass/potential TFs, calculated with the removal of the cation contribution [54,55,61]. Suppressed loops in the upper-right quadrant are always observed, characteristic of the contribution of anions and solvent molecules exchanged in the same direction and magnified due to the removal of the contribution of the cations.

#### 3.3.2. Kinetic Parameters and Transfer Resistance

**Figure 5:**
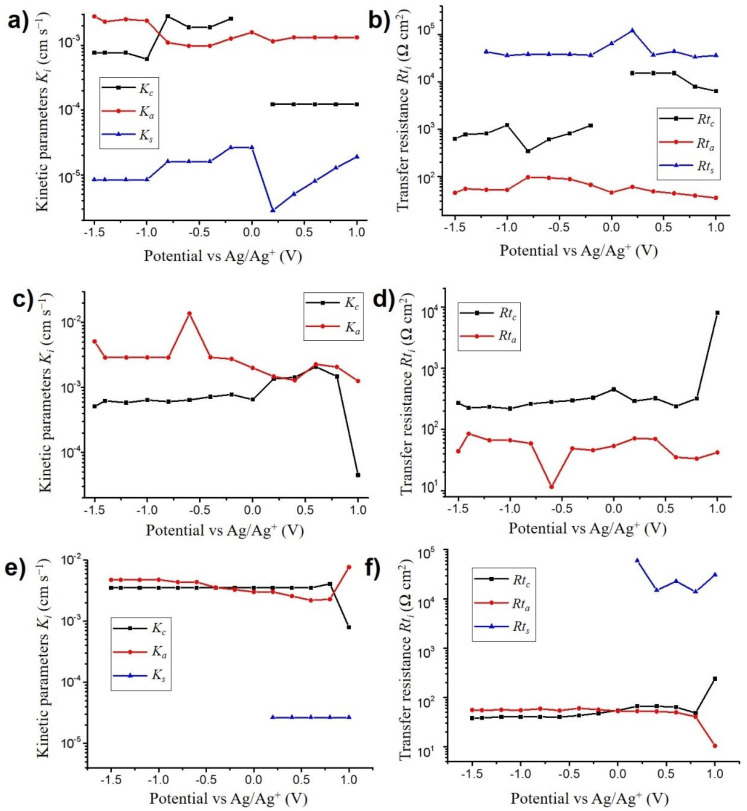
Evolution of the kinetic parameters *K_i_* (cm s^−1^) (**a**,**c**,**e**) and the transfer resistance values *Rt_i_* (Ω cm^2^) (**b**,**d**,**e**) in (**a**,**b**) PC/TBAClO_4_, (**c**,**d**) PC/LiClO_4_ and (**e,f**) PC/N_1114_TFSI.

For each studied electrolyte and at each measured potential, the fitted ac-electrogravimetry data allowed the derivation of the kinetic and conductance parameters *K_i_* and *G_i_* of each species in the model to be obtained. Figure 5a,c,e depict the evolution of the kinetic parameters, *K_i_*_,_ obtained for each electrolyte. For the TBAClO_4_ case, anions and cations have equivalent values of kinetic parameters around 10^−3^ cm s^−1^, showing that ClO_4_^−^ anions are exchanged as fast as the heavier TBA^+^ (*M_TBA_^+^* = 242.5 g mol^−1^) below 0 V vs. Ag/Ag^+^ (Figure 5a). Above 0 V, ClO_4_ anions are also exchanged faster with *K_a_* = 10^−3^ and *K_c_* = 10^−4^ cm s^−1^. This is not the case for LiClO_4_, where ClO_4_^−^ anions (*K_a_* = 3 × 10^−3^ cm s^−1^) are exchanged faster than monosolvated Li^+^ cations (*K_c_* = 6 × 10^−4^ cm s^−1^) (Figure 5c). It is noted that the ClO_4_^−^ anions are slightly faster in PC/LiClO_4_ than that occurs in PC/TBAClO_4_, indicating the influence of the ion pairs on the interfacial kinetics.

In the case of the diluted N_1114_TFSI ionic liquid, both cations and anions have their kinetic parameters around 3·10^−3^ cm s^−1^, showing that massive anions such as TFSI^−^ (*M_TFSI_^-^* = 280 g mol^−1^) can be exchanged as rapidly as the lighter ClO_4_^−^ anions (Figure 5e). For all electrolytes with free solvent molecule exchanges in the model, these take place at very slow rates of transfer (*K_s_* = 2 × 10^−6^ to 5 × 10^−6^ cm s^−1^). From these results, it appears that the ion size has no major effect on the exchange kinetics.

The transfer resistance $Rt_{i}=\frac{1}{FG_{i}}$ is depicted for each electrolyte in Figure 5b,d,f at all the studied potentials. Free solvent molecules always have the highest transfer resistance (*Rt_s_* = 13 to 120 kΩ cm^2^), followed by cations (*Rt_(TBA_^+^_)_* = 0.4 to 15 kΩ cm^2^, *Rt_(Li_^+^_PC)_* = 0.2 to 0.5 kΩ cm^2^, *Rt_(N1114_^+^_)_* = 40 to 250 Ω cm^2^) and anions (*Rt_(ClO4_^−^_)_* = 10 to 100 Ω cm^2^, *Rt_(TFSI_^−^_)_* = 10 to 60 Ω cm^2^). The combination of *K_i_* and *Rt_i_* collected for each species at various potentials reveals the dynamics of the charge transfer mechanism, showing that in many cases anions have a major contribution to the energy stored in VOGN electrodes.

#### 3.3.3. Concentration and Mass Variations for Each Species

The values of *K_i_* and *G_i_* also allow the calculation of the relative concentration changes of individual species, *C_i_–C*_0_*,* at the electrode, through the integration of the Equation (3), leading to Equation (4) as follows [53,54,55]:

(3) $$\frac{\Delta C_{i}}{\Delta E}(\omega )=\frac{-G_{i}}{j\omega d_{f}+K_{i}}$$

(4) $${C_{i}-C_{0}=\int_{E_{0}}^{E_{i}}\frac{\Delta C_{i}}{\Delta E}(\omega )\;dE|}_{\omega \to 0}=\;\;\int_{E_{0}}^{E_{i}}\frac{-G_{i}}{K_{i}}dE$$

**Figure 6:**
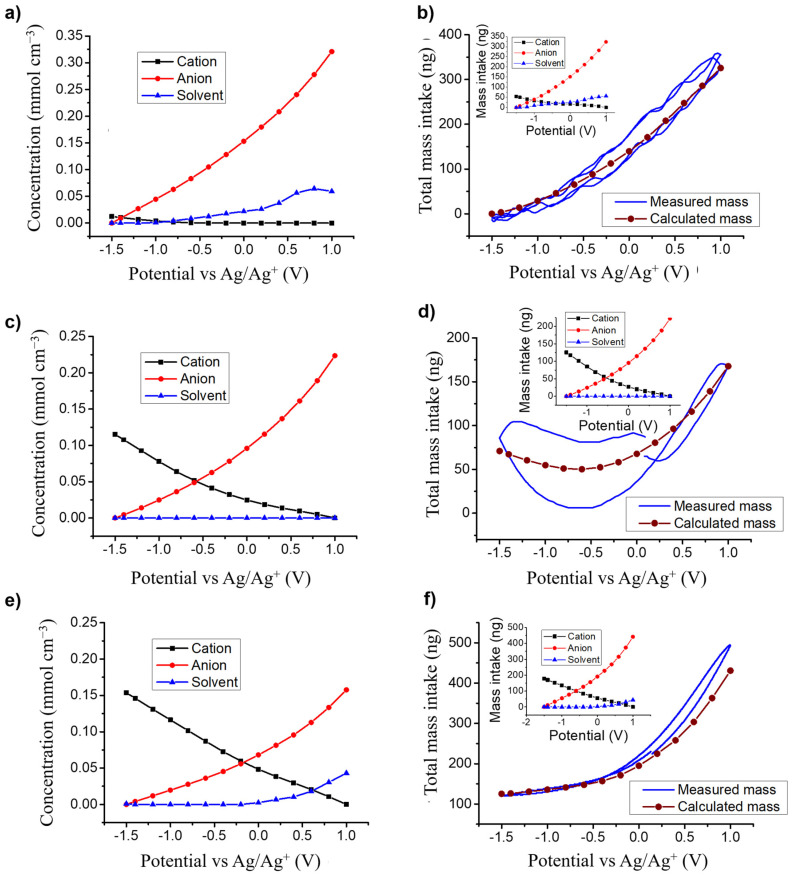
Evolution of the relative concentrations, *C**_i_**–C*_0_, of each species exchanged, derived from ac-electrogravimetric measurements (**a**,**c**,**e**) and the resulting total mass variations calculated from ac-electrogravimetry and measured with EQCM at 10 mV s^−1^ (results for individual species as inset) (**d**,**e**,**f**) in (**a**,**b**) PC/TBAClO_4_, (**c**,**d**) PC/LiClO_4_ and (**e**,**f**) PC/N_1114_TFSI.

Figure 6a,c,e depict *C_i_–C*_0_ calculated for each electrolyte and for each species identified in the corresponding theoretical model to fit the ac-electrogravimetry data. In PC:TBAClO_4_, anions have the highest concentration variations, with a linear increase from −1.5 to 1 V vs. Ag/Ag^+^ while the concentration of TBA^+^ cations rises very slightly at negative potentials (−0.75 to −1.5 V vs. Ag/Ag^+^) (Figure 6a). Thus, a permselectivity of the electrode/electrolyte interface, meaning that practically only one species dominates the ionic exchanges, is observed. These results show that ClO_4_^−^ anions behave almost in the same way when the VOGN electrode is charged and discharged: We have a permselectivity of anions in both cases due to the larger size of TBA^+^ cations except for a narrow permselectivity failure zone at low potentials. In the case of LiClO_4_, both ClO_4_^−^ anions and monosolvated Li^+^ cations present high concentration variations with around 25 mmol cm^−3^ for anions and 13 mmol cm^−3^ for cations over the whole potential window (Figure 6c). This clearly shows that reducing the cation size can change the overall exchange mechanisms, where in this case both cations and anions can be exchanged at the same time. For the mixture of PC and N_1114_TFSI, both cations and anions have the same magnitude of concentration changes in opposite directions (Figure 6e). This demonstrates that both species are exchanged symmetrically in the studied potential window, even though the anion’s larger mass (M_TFSI_^-^ = 280 g mol^−1^) compared to cations (M_N1114_^+^ = 116 g mol^−1^) dominated the classical EQCM measurements in terms of mass variations. Thus, for these two last electrolytes multiple species are exchanged, exhibiting a failure of the electrode’s permselectivity.

Using the concentration versus potential profiles in Figure 6, the potential-induced mass variations (*m_i_*) of the VOGN electrode can then be calculated (*m_i_* = *M_i_* *× V_electrode_ ×* (*C_i_–C*_0_), with *M_i_* the molar mass of species *i*, *i*: cation, anion or free solvent molecule and *V_electrode_* is the electrode volume). Figure 6b,d,f depict the total mass variations measured during EQCM and calculated with $m=\sum m_{i}$ for each electrolyte, with the individual calculated mass variations *m_i_* depicted in the inset. In each case, Δ*m*_total_ calculated from ac-electrogravimetry is in good agreement with the global Δ*m* given by EQCM, further validating the models used to fit the ac-electrogravimetry data and complementarity of the two electrogravimetric methods.

## 4. Discussion

The energy storage mechanisms at the electrode–electrolyte interface of VOGNs with various electrolytes (TBAClO_4_, LiClO_4_ and N_1114_TFSI) have been investigated by using EQCM and ac-electrogravimetry methods. The deconvolution of the global response of EQCM into distinct contributions and the kinetics information on the species electroadsorbed brought significant insights concerning the role of ionic exchanges (anions, cations, solvation numbers and free solvent contribution) on the capacitive properties of VOGNs, which are summarized as follows:

(a) VOGNs-PC/TBAClO_4_: A major contribution of ClO_4_^−^ anions was found that they are faster than their corresponding cation (TBA^+^) counterparts at most of the potentials, as well as at higher concentration variations along the whole electrochemical window (−1.5 to 1 V). Consequently, an anion electro-adsorption process was identified to be the most predominant energy storage mechanism, while cations and free solvent molecules are given non-negligible supporting roles. This tendency was found to be similar to PC/TBABF_4_ already examined through similar electrogravimetric methods [45].
(b) VOGNs-PC/LiClO_4_: The nature of the cation greatly affects the electrochemical behavior as evidenced in the particular case of PC/LiClO_4_. Monosolvated Li^+^ cations (PC-Li^+^) and ClO_4_^−^ anions were found to play key roles in the energy storage mechanism of VOGNs. Thus, solvated Li^+^ cations predominates at negative potentials (−1.5 to −0.5 V) as shown by its higher concentration, whereas ClO_4_^−^ is more predominant at positive potentials. A V-shape gravimetric response was obtained in this case.
(c) VOGNs-PC/N_1114_TFSI: The high deviation of MPE values compared to the theoretical molar mass of N_1114_^+^ and TFSI^−^ showed that anions are not the only species exchanged on the energy storage mechanism of VOGN. The ac-electrogravimetry allowed unveiling that N_1114_^+^ cations and TFSI^−^ anions, with an addition of PC molecules, play a key role. More specifically, both cations and anions exhibited similar kinetic parameters and transfer resistance values, as well as the same magnitude of concentration variations in the studied potential window. Consequently, both species contributed equally to the capacitive properties of VOGNs.
(d) The areal capacitance values obtained by varying the nature of the electrolyte have shown no significant difference in spite of the change in size and the molar mass of the anions and cations. However, the VOGNs-PC/TBAClO_4_ electrode/electrolyte configuration shows a good permselectivity behavior in the larger potential window and for this reason can be recommended, among the others investigated in this work.

In a classical organic electrolyte, i.e., without ILs, the main contribution depends on the cation nature/size. Keeping the same anion but changing the cation size leads to the following: (i) in the presence of a poorly solvated large size cation, the anion contribution is predominant; (ii) while with a small cation, a mix between the cation/anion contributions is observed depending on the applied potential. In the case of a composite electrolyte, mix between IL and organic solvent, each ion, coming from the IL, plays an equivalent role in terms of concentration changes in the VOGN structure. In this case, the higher mass change was observed in the potential range of exploration and due to the high molar mass of the anion of the IL, the final mass change can be considered as a purely anion transfer. Only the ac-electrogravimetric explorations allow these electrochemical subtleties, occurring during the charge compensation process, to be discriminative without any doubt.

Overall, the present results in this work shed a new light on the comprehension of energy storage mechanisms of VOGN supercapacitors and the judicious choice of electrolyte compositions by using electrogravimetry-based methods. The importance of the ionic exchanges at the electrode–electrolyte interface is vital to unravel a better design of high-performance supercapacitor devices in the coming years. In addition, this study also paves the way to explore new perspectives in the field of Li-ion batteries, regarding critical aspects as for example the formation of the solid electrolyte interface.

## Figures and Tables

**Table 1:** Values obtained for the resonant frequency (*f*), peak width at half height (*W*), motional resistance (*Rm*), and quality factor (*Q*), for a GaPO_4_ resonator with Au electrodes in air and PC, as well as before and after VOGN growth.

System	Fluid Viscosity *η* (mPa s)	Fluid Density *ρ* (kg m^−3^)	Resonant Frequency *f* (Hz)	Peak Width *W* (Hz)	Motional Resistance, *Rm* (Ω)	Quality Factor, *Q*
Au in air	0.0184	1.17	5,830,000 (±15)	700 (±27)	33 (±5)	8300 (±135)
Au in PC	2.5	1200	5,827,000 (±25)	5046 (±64)	188 (±8)	1155 (±85)
VOGNs in air	0.0184	1.17	5,828,000 (±17)	756 (±25)	24 (±6)	7700 (±120)
VOGNs in PC	2.5	1200	5,819,000 (±27)	5726 (±72)	229 (±12)	1016 (±70)

**Table 2:** Mass per mole of electrons (MPE) values obtained for the different electrolytes (TBAClO_4_, LiClO_4_ and PC/N_1114_TFSI) from the electrochemical quartz crystal microbalance (EQCM) data. The corresponding anion and cation molar masses are also given.

Electrolyte	MPE_(experimental)_ (g mol^−1^)	Anion Mass (g mol^−1^)	Cation Mass (g mol^−1^)
TBAClO_4_	70 (above −1 V) 30 (below −1 V)	99.4	242
LiClO_4_	34 (above −0.75 V) −31 (below −0.75 V)	99.4	6.9
PC/N_1114_TFSI	90 (above 0 V) 25 (below −0.3 V)	280.1	106
